# CNN-Based Volume Flow Rate Prediction of Oil–Gas–Water Three-Phase Intermittent Flow from Multiple Sensors

**DOI:** 10.3390/s21041245

**Published:** 2021-02-10

**Authors:** Jinku Li, Delin Hu, Wei Chen, Yi Li, Maomao Zhang, Lihui Peng

**Affiliations:** 1 Department of Automation, Tsinghua University, Beijing 100084, China; li-jk15@mails.tsinghua.edu.cn (J.L.); wchen16@mails.tsinghua.edu.cn (W.C.); 2 School of Engineering, The University of Edinburgh, Edinburgh EH9 3JW, UK; delin.hu@ed.ac.uk; 3 Tsinghua Shenzhen International Graduate School, Tsinghua University, Shenzhen 518055, China; liyi@sz.tsinghua.edu.cn (Y.L.); zhangmaomao@sz.tsinghua.edu.cn (M.Z.)

**Keywords:** CNN, volume flow rate, three-phase intermittent flow, multiple sensors

## Abstract

In this paper, we propose a deep-learning-based method using a convolutional neural network (CNN) to predict the volume flow rates of individual phases in the oil–gas–water three-phase intermittent flow simultaneously by analyzing the measurement data from multiple sensors, including a temperature sensor, a pressure sensor, a Venturi tube and a microwave sensor. To build datasets, a series of experiments for the oil–gas–water three-phase intermittent flow in a horizontal pipe, in which gas volume fraction and water-in-liquid ratio ranges are 23.77–94.45% and 14.95–86.97%, respectively, and gas flow superficial velocity and liquid flow superficial velocity ranges are 0.66–5.23 and 0.27–2.14 m/s, respectively, have been carried out on a test loop pipeline. The preliminary results indicate that the model can provide relative prediction errors on the testing-1 dataset for the volume flow rates of oil-phase, gas-phase and water-phase within ±10% with 94.49%, 92.56% and 95.71% confidence levels, respectively. Additionally, the prediction results on the testing-2 dataset also demonstrate the generalization ability of the model. The consuming time of a prediction with one sample is 0.43 s on an Intel Xeon CPU E5-2678 v3, and 0.01 s on an NVIDIA GeForce GTX 1080 Ti GPU. Hence, the proposed CNN-based prediction model, which can fulfill the real-time application requirements in the petroleum industry, reveals the potential of using deep learning to obtain accurate results in the multiphase flow measurement field.

## 1. Introduction

Multiphase flow is mixture flow of different components or phases, which widely exist in many industry fields, such as oil field exploitation, chemical engineering, and the metallurgical industry. The flow rate measurement of multiphase flow is very important and helpful for process monitoring and optimizing. An important example of this is the accurate measurement of outcomes including oil, gas, and water for an individual well in the petroleum industry, which is crucial for the custody transfer and the digital management of an oil field [1]. However, due to the complex flow characterizations and harsh field conditions, it has long been a challenge to accurately measure the flow rate of multiphase flows in industry.

Since the 1980s, for flow rate prediction, many multiphase flow meters using different techniques have been developed [2]. In the most conventional multiphase flow rate measurement techniques, a separator and some single-phase flow meters are needed. This indirect-measuring method can achieve relatively high precision. Nevertheless, the separator is bulky, and this method is costly and has a poor real-time ability [1]. Hence, it is significant to find ways that are cost-effective and convenient to directly obtain the flow rate of the multiphase flow.

For realizing direct flow rate measuring, many researchers have tried a variety of methods based on various theories. In 2005, Z. Huang and D. Xie et al. combined a Venturi meter and an electrical capacitance tomography (ECT) system to measure the flow rate of gas–oil two-phase flow [3]. In 2009, P. Mehdizadeh, D. Farchy and J. Suarez combined the Coriolis flow meter and the microwave-based water cut meter to measure the volume flow rate of each phase [4]. In 2012, C. Xie and Z. Wu estimated the liquid volume flow rate of multiphase flow by utilizing the microwave Doppler system [5]. In 2014, F. Zhou, M. Henry and M. Tombs combined Coriolis mass flow rate metering and water cut metering to obtain the mass flow rates of oil, gas and water, respectively, without phase separation [6]. In 2015, C. Yuan and Y. Xu et al. used dimensionless analysis to investigate the gas flow rate measurement for wet gas with the Venturi tube [7]. Currently, there are certain commercialized multiphase flow meters (MPFMs) applied practically, which usually combine various measurement sensors and measurement techniques [8,9,10,11]. These multiphase flow meters are usually very expensive and perform well under the condition of high liquid-phase production, greater than several hundred cubic meters per day. For example, the typical measurement uncertainty on liquid and gas flow rates of Roxar MPFM 2600 M is 10% with a 95% confidence level, while working under the conditions of 15–85% GVF and total flow velocity range of 5–25 m/s [8]. It is difficult to commonly apply these flow meters for the measurement of an individual well with low liquid-phase production, which is the majority of onshore oilfields in China. Thus, the development of cost-effective flow meters and measurement techniques for oil–gas–water three-phase flow is still a big challenge and an open problem. Meanwhile, the aforementioned measurement methods are generally based on the empirical or semi-empirical model. Although they have been developed and obtained good measurement results, there is still much room to improve their performances for practical applications. Hence, new data analytics has to be developed.

In recent years, machine learning, especially deep learning based upon various artificial neural networks, as a data-driven method, has gradually become a very hot research topic and attracted attention in the multiphase-flow-measurement society. In 2014, S. Fan and T. Yan presented a method based on a multilayer feedforward neural network and conductance probes to obtain the gas and liquid flow rates of an air–water two-phase slug flow [12]. In 2015, J. Chen and L. Xu et al. measured the water cut of an oil–water two-phase flow on the basis of support vector regression (SVR) by combining the total flow rate and the response of a conductance probe [13]. In 2016, S. Azizi, E. Ahmadloo and M. M. Awad predicted the void fraction of a gas–liquid two-phase flow using multilayer perceptron neural networks with the pipe inclination angle and the superficial Reynolds number of gas and liquid as the input parameters [14]. In 2019, B. Bahrami and S. Mohsenpour et al. used the feed-forward neural network approach together with the flow parameters and the statistical characteristics of pressure signals to estimate the flow rates of individual phases in the oil–gas–water multiphase flow [15]. In addition, the applications of convolutional neural networks have been studied [16,17,18]. With the help of the powerful representational capacity of machine learning, measurement methods combined with machine learning have obtained promising achievements.

In the past few years, deep learning has achieved great successes in many fields [19]. The theory related to deep learning has also made great progress, including the studies about different neural network architectures, the more powerful computation ability of GPUs, big data, the various training techniques and open-source deep-learning platforms, etc. Hence, it is worthy to explore the measurement method by using the prevailing deep-learning techniques to implement the black-box soft-sensing of complex systems [20,21,22]. For the real-time measurement and digital management of an individual well in the petroleum industry, there has been an increasing need to realize the flow rate measurement of the multiphase flow in convenient, cost-effective and accuracy-assured ways. From the perspective of combining deep learning with the petroleum industry, this paper is trying to explore the data-driven black-box soft-sensing methods for complex systems such as an oil–gas–water three-phase intermittent flow by using recently booming deep learning techniques to analyze the measured data from cost-effective sensors, which may bring new data analytics into the multiphase flow measurement and extend the application of deep learning.

In this paper, based on the convolutional neural network (CNN), the volume flow rate prediction model for the oil–gas–water three-phase intermittent flow is proposed to organically integrate the measurement data from the multiple sensors which are mounted in the horizontal pipe of our experimental setup. For this, we use the temperature sensor and the pressure sensor to obtain the flow temperature and the pipeline pressure, respectively, and use the Venturi tube to obtain the differential pressures. Similar to many commercial MPFMs using radioactive methods [9,10,11], we utilize the microwave sensor to obtain the data which are sensitive to the phase fraction. Through analyzing the measurement data, the CNN-based model can predict the oil-phase volume flow rate (OVFR), the gas-phase volume flow rate (GVFR) and the water-phase volume flow rate (WVFR) simultaneously.

In the rest of this paper, we present our model and experiments in detail. Section 2 introduces our experimental system and working conditions. Section 3 presents the proposed CNN-based prediction model in detail. In Section 4, the obtained experimental results are discussed. Finally, we conclude in Section 5.

## 2. Experimental System and Working Conditions

**Figure 1:**
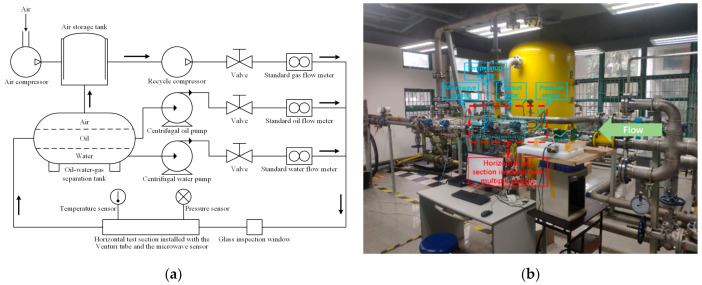
The schematic diagram and picture of the experimental system. (**a**) The schematic diagram; (**b**) The picture.

The schematic diagram of the experimental system in our multiphase laboratory is shown as Figure 1a and the corresponding picture is shown as Figure 1b. In this test loop with 50 mm diameter, which mimics the real pipeline of an individual well, the oil-phase medium is white oil, the gas-phase medium is air and the water-phase medium is tap water. The distance of the center sections between the Venturi tube and the microwave sensor is 560 mm, which is about 10 times that of the pipe diameter. The Venturi tube is located upstream of the microwave sensor.

Firstly, the air compressor supplies the static pressure for the whole experimental system. Then, the air from the air storage tank is pumped into the pipeline of our experimental apparatus by the recycle compressor. In the separation tank, when the liquid-phase flow rate and the gas-phase flow rate in working conditions are less than 20 and 150 m^3^/h respectively, the oil–gas–water three-phase flow will sedimentate as single-phase air, oil and water quickly due to their different densities, which satisfies our experimental requirements. The oil and water are pumped into the pipeline by the centrifugal pumps, respectively. These three single-phase fluids flow through the valves and standard flow meters. These valves can control the individual flow rate of each single-phase component. The standard flow meters can provide the reference volume flow rates of each single-phase flow. Afterwards, air, oil and water are mixed into one pipe. Then, the oil–gas–water three-phase flow passes through the glass inspection window and the horizontal test section installed with multiple sensors. Finally, the three-phase flow goes back into the separation tank.

**Figure 2:**
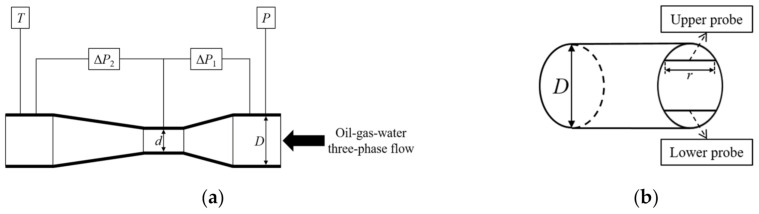
The schematic diagrams of the multiple sensors. (**a**) The structure diagram of the section installed with the temperature sensor, the pressure sensor and the Venturi tube; (**b**) The structure diagram of the microwave sensor.

Figure 2 shows the structure diagrams of the multiple sensors installed on the horizontal test section of Figure 1. There are four types of sensor, which are the Venturi tube and the microwave sensor supplemented with the temperature sensor and the pressure sensor. The upstream and downstream pipe diameters of the test section are both 50 mm. In Figure 2a,b, the upstream diameter *D* and the throat diameter *d* of the Venturi tube are 50 and 25 mm, respectively. There are two probes in the microwave sensor, whose lengths *r* are both 20 mm. From the upper probe, the upper microwave phase and amplitude can be acquired, and correspondingly, from the lower probe, the lower microwave phase and amplitude can be acquired. Hence, the multiple sensors can provide eight types of sensor reading, i.e., Δ*P*_1_ (the differential pressure of the convergent section) and Δ*P*_2_ (the differential pressure of the divergent section) from the Venturi tube, *β*_1_ (the upper microwave phase), *A*_1_ (the upper microwave amplitude), *β*_2_ (the lower microwave phase) and *A*_2_ (the lower microwave amplitude) from the microwave sensor, *T* (the flow temperature) from the temperature sensor and *P* (the pipeline pressure) from the pressure sensor.

Table 1 shows the standard flow meters used in our experiments. The GVF and WLR are calculated via the equations as follows

(1) $$\mathrm{GVF}=\frac{Q_{g}}{Q_{o}+Q_{g}+Q_{w}}\cdot 100\%,$$

(2) $$\mathrm{WLR}=\frac{Q_{w}}{Q_{o}+Q_{w}}\cdot 100\%,$$

where *Q_o_*, *Q_g_* and *Q_w_* are the reference volume flow rates of the oil, gas and water, which are measured by the standard flow meters listed in Table 1, respectively.

**Figure 3:**
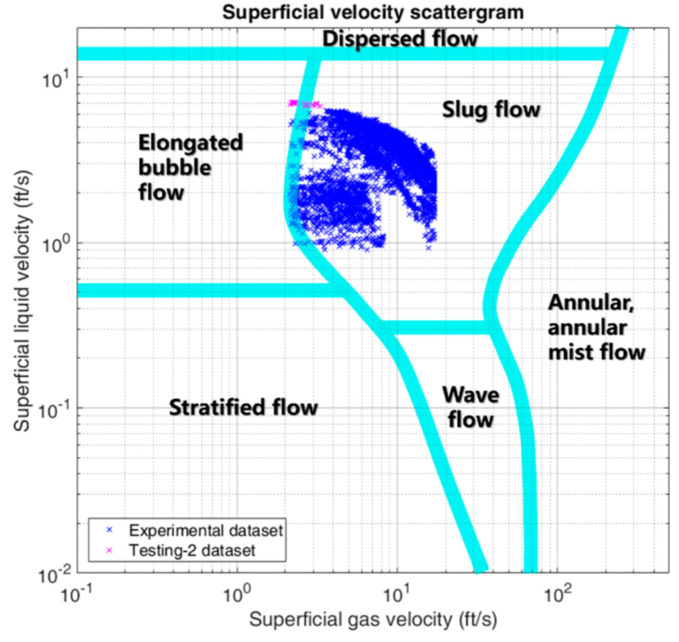
The flow regime map.

The working conditions of our experiments are shown in Table 2, where *T*, *P*, Δ*P*_1_, Δ*P*_2_, *β*_1_, *A*_1_, *β*_2_ and *A*_2_ have the same meaning as mentioned above. As aforementioned, certain commercialized multiphase flow meters perform well under the condition of high liquid-phase production. In the study of this paper, the proposed CNN-based method can be applied to the flow condition of low liquid-phase production, which is consistent with most well-head oil–gas–water three-phase flows in the onshore oilfields in China. A series of experiments under the working conditions described in Table 2 are carried out on the test loop and the related data are collected. These collected data are used to train, validate, and demonstrate the performances of our proposed CNN-based prediction model. According to [23,24], we can obtain the flow regime map inside the horizontal pipeline by using the superficial velocities of gas-phase and liquid-phase and plotting the superficial flow velocity scattergram in Figure 3, wherein, the blue points represent the scattergram in accordance with the experimental dataset, which will be split into the training dataset, the validation dataset and the testing-1 dataset, and the magenta points represent the scattergram in accordance with the testing-2 dataset. The testing-2 dataset has 70 samples, whose flow rate range is out of range regarding the experimental dataset in the blue color. The testing-2 dataset is used to verify the generalization ability of the proposed model and will be discussed in Section 4. From the flow regime map, it can be found that there is a mass of slug flow and a little elongated bubble flow which are collectively called the intermittent flow, which is consistent with the observations from the glass inspection window.

## 3. Prediction Model Based on CNN

Artificial neural networks (ANNs) are computational models that are loosely inspired by the neurosciences and are mainly composed of simple processing units and their interconnections, so ANNs can learn from experience through modifying these interconnections [25]. Various ANN models have been studied in the past and recently researched in different applications. In [18], X. Lin and H. Wang et al. analyzed the relationship between the WLR and the microwave data (i.e. *β*_1_, *A*_1_, *β*_2_ and *A*_2_), and, by combining the microwave sensor with the Venturi tube, they proposed a dual convolutional neural network model to predict the volume flow rates of the oil and water in the oil–gas–water multiphase flow. From [18], we can obtain the following relationship

(3) $$\left({\hat{Q}}_{o},{\hat{Q}}_{w}\right)=f\left(P,\Delta P_{1},\Delta P_{2},\beta_{1},A_{1},\beta_{2},A_{2}\right),$$

where ${\hat{Q}}_{o}$ and ${\hat{Q}}_{w}$ represent the predicted OVFR and the predicted WVFR, respectively, and *P*, Δ*P*_1_, Δ*P*_2_, *β*_1_, *A*_1_, *β*_2_ and *A*_2_ are the sampled sensor data have the aforementioned meanings. From [18,26,27], the predicted gas-phase volume flow rate can be calculated by the equation shown below, in the traditional method with the Venturi tube

(4) $${\hat{Q}}_{g}=\frac{C_{\varepsilon }}{\sqrt{1-\beta^{4}}}\times \frac{\pi }{4}\beta^{2}D^{2}\times \sqrt{\frac{2\Delta P_{g}}{\rho_{g}}},$$

where *C* is the discharge coefficient, *ε* is the expansion factor, *β* is the beta ratio, *D* is the pipe internal diameter, Δ*P_g_* is the differential pressure measured by the Venturi tube and *ρ_g_* is the gas-phase density. From this equation, we can know that, besides the differential pressure, the gas-phase volume flow rate is also affected by the gas-phase density *ρ_g_* in working conditions, which is dependent on *T* and *P*. Hence, we need the flow temperature *T* as an input parameter to further predict the gas-phase volume flow rate. Combining Equations (3) and (4), we can obtain the relationship as

(5) $$\left({\hat{Q}}_{o},{\hat{Q}}_{g},{\hat{Q}}_{w}\right)=f\left(T,P,\Delta P_{1},\Delta P_{2},\beta_{1},A_{1},\beta_{2},A_{2}\right),$$

where *f*(·) denotes the prediction model that we focus on.

In this section, the CNN-based regression model is presented to predict the volume flow rates of oil-phase, gas-phase and water-phase simultaneously. Eight types of measurement data, which are *T*, *P*, Δ*P*_1_, Δ*P*_2_, *β*_1_, *A*_1_, *β*_2_ and *A*_2_, are used as the input in our model. OVFR, GVFR and WVFR is the output. To clearly depict the model, we define ${\hat{Q}}_{o}$, ${\hat{Q}}_{g}$, ${\hat{Q}}_{w}$, *Q_o_*, *Q_g_* and *Q_w_* as representing the predicted OVFR, the predicted GVFR, the predicted WVFR, the reference OVFR, the reference GVFR and the reference WVFR, respectively.

### 3.1. Data Preprocessing

The CNN-based measurement model has to be trained by using the experimental data before it is deployed for prediction. The sampling frequency of multiple sensors is around 10 Hz. Each point in the flow regime map shown as Figure 3 corresponds to one specific working condition described as Table 2, which lasted about 3 min and resulted in a group of time series of experimental data composed of 1800 sampling points of *P*, Δ*P*_1_, Δ*P*_2_, *T*, *β*_1_, *A*_1_, *β*_2_ and *A*_2_. The reference volume flow rates corresponding to this 3-minute period are the calculated mean values of readings from the standard flow meters. Hence, the model’s input is a data matrix in 1800 by 8, which means a data group including 1800 sampling points of eight sensor readings (i.e., *P*, Δ*P*_1_, Δ*P*_2_, *T*, *β*_1_, *A*_1_, *β*_2_ and *A*_2_). The feature extraction and flow rate prediction can be implemented by CNN. The model’s output is the predicted OVFR (${\hat{Q}}_{o}$), the predicted WVFR (${\hat{Q}}_{w}$) and the predicted GVFR (${\hat{Q}}_{g}$).

**Figure 4:**
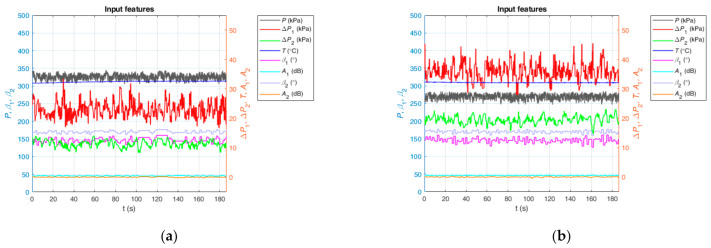
Two data samples of different working conditions. (**a**) Corresponding reference volume flow rates: *Q_o_* = 5.10 m^3^/h, *Q_g_* = 17.02 m^3^/h, *Q_w_* = 2.90 m^3^/h; (**b**) Corresponding reference volume flow rates: *Q_o_* = 4.86 m^3^/h, *Q_g_* = 28.54 m^3^/h, *Q_w_* = 3.90 m^3^/h.

In our model, the input data are a group of time series obtained from the real multiple sensors. Hence, we need to implement data cleaning for the collected data. Firstly, we compensated and corrected all of the sensor data. Then, we dropped the input data groups including measuring points out of the measuring range of sensors and the corresponding target data (i.e., *Q_o_*, *Q_g_* and *Q_w_*). Finally, we shuffled these reserved input and corresponding target data and then partitioned them into the different datasets we need. After data cleaning, we obtained 8177 samples as the training dataset, 1100 samples as the validation dataset, 1560 samples as the testing-1 dataset and 70 samples as the testing-2 dataset. Figure 4 shows two data samples, which correspond to two different working conditions. They are the time diagrams of the input features. There are two vertical axes, of which the left corresponds to *P*, *β*_1_ and *β*_2_, and the right corresponds to Δ*P*_1_, Δ*P*_2_, *T*, *A*_1_ and *A*_2_ to show the curves clearly. In Figure 4a, the corresponding *Q_o_*, *Q_g_* and *Q_w_* are 5.10, 17.02 and 2.90 m^3^/h, respectively. In Figure 4b, the corresponding *Q_o_*, *Q_g_* and *Q_w_* are 4.86, 28.54 and 3.90 m^3^/h, respectively. From Figure 4, it is noteworthy that the relatively evident differences in input features between Figure 4a,b show that it is feasible to learn the underlying flowrate-sensitive features from inputs using deep learning techniques.

### 3.2. CNN-Based Prediction Model

**Figure 5:**
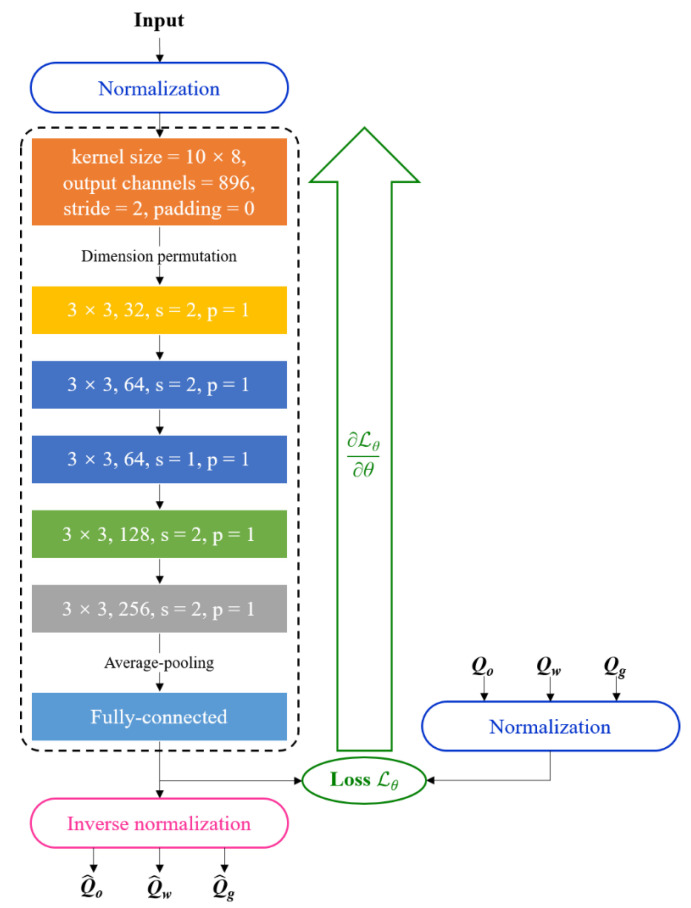
The architecture diagram of the convolutional neural network (CNN)-7-based prediction model for the volume flow rates of the individual phases.

Convolutional neural networks (CNNs) are networks used to process data which have a known, grid-like topology [19]. In recent years, CNNs have been developed rapidly and various novel CNN architectures have emerged. Nowadays, CNNs are widely used in various practical scenarios, like image classification [28] and sentence classification [29,30], and have made tremendous achievements. In this paper, we propose a CNN-based prediction model for the volume flow rates of the oil-phase, the gas-phase and the water-phase, whose architecture is depicted as Figure 5. In the proposed model, the volume flow rates of individual phases are obtained simultaneously. Through the trial and evaluation about convolutional neural networks with different depths, which vary from 2 to 16 layers, we determine that the CNN-based prediction model has the structure of seven layers, i.e., the CNN-7 with the structure of Figure 5 for the volume flow rate predictions.

As illustrated in Figure 5, a group of input features are fed into the multiple convolutional layers. Then, the features extracted by convolutional layers are reshaped and pass through the final subsequent fully connected layer to obtain the prediction of OVFR, WVFR and GVFR. In our proposed architecture, the operation of the first convolutional layer is 1D convolution, and the operations of the other convolutional layers are 2D convolutions. Hence, there is a dimension permutation operation of intermediate features between the first layer and the second layer.

From Figure 5, it can be noted that there are normalization operations for the raw input and target data to train the model more effectively. In this paper, each type of input data and target data is normalized into [−1, 1] and [0, 1] via rescaling. Inverse normalization should be applied to restore the outputs to the scale of the original target data to obtain ${\hat{Q}}_{o}$, ${\hat{Q}}_{w}$ and ${\hat{Q}}_{g}$ [31,32]. In Figure 5, the model part inside the dashed box is the structure, including all trainable parameters of the model, which are updated by the back-propagation.

Table 3 shows some details of the CNN-7 architecture. In this table, “BN” means the batch normalization and “ReLU” is the applied activation function [19]. The output shape is represented by “channels of feature map × height of feature map × width of feature map”. Note that, in Table 3, there are six convolutional layers (CONV1~CONV6) and a fully connected layer (FC).

References [17,18] both leverage the CNN architecture to realize the flow rate prediction for the multiphase flow. However, the research and the proposed method in this paper are different from those in [17,18]. Compared with [18], the proposed CNN-7-based model has the following differences and novelties. Firstly, besides the flow rates of the oil and water, the CNN-7-based model can obtain the gas flow rate in the oil–gas–water three-phase flow. Secondly, the CNN-7-based model is combined with ensemble learning to improve the prediction performance, which is explained in detail in Section 3.3. Thirdly, the proposed CNN-7-based model is more compact and concise. The CNN-7-based model in this paper uses both 1D convolution and 2D convolution operations to extract better features, and the input parameters are fed into one convolutional neural network so that these input parameters are better coupled to obtain better results, which will be discussed in Section 4. Finally, on the basis of the supervised-learning-based model studied in this paper, a novel machine-learning-based data analytics framework for the measurement of multiphase flow is proposed, which will be discussed in Section 4.

In [17], CNN is used to predict the oil flow rate, the gas flow rate and GVF in the gas–oil two-phase flow through analyzing the flow patterns before and after the Venturi tube, which are obtained from dual electrical capacitance tomography sensors and a linear back-projection algorithm. Hence, it can be seen that the research goal and method in [17] are very different from those in this paper. In addition, the CNN model in [17] is derived from the Inception V3 model, which is much more complicated than the proposed CNN-7-based model in this paper.

### 3.3. Loss Function and Ensemble Learning

As previously mentioned, we have demonstrated the CNN-7 prediction model in detail. In addition to the architecture, the design of the loss function, which is suitable for our application scenario, is also very crucial. The prediction of OVFR, GVFR and WVFR is actually a regression problem. Therefore, we utilize the mean squared error (MSE) as the loss function [19]

(6) $$L(\theta )=E_{\left(x,y)∼p_{data}(x,y\right)}\left\{∥y-f(x;\theta ){∥}_{2}^{2}\right\},$$

where ***x*** denotes a group of normalized input features; ***y*** denotes the corresponding normalized reference vector; ***θ*** denotes the trainable parameters of the model; *f*(·) rep-resents the model part including ***θ***, shown in the dashed box of Figure 5. The training process of the CNN-based measurement model is used to resolve the optimization problem as bellow

(7) $$\theta^{*}=\underset{\theta }{\arg\min}L(\theta ),$$

where θ^*^ represents the well-trained parameters. In deep learning, the trainable parameters are generally updated by the back-propagation algorithm. We selected the Adam algorithm to train our model, which is a method for stochastic optimization about back-propagation [33].

To improve the predictive effects further, we combined the trained CNN-based model with the inspiration of ensemble learning. Ensemble learning is a method which constructs multiple individual learners and combines them to achieve the learning task [34]. We trained the CNN-7-based prediction model for multiple trials and the trained model of each trial is an individual learner. Then, we used the averaging combination scheme to combine the learners to obtain the final prediction results. The applied averaging combination scheme can be demonstrated as follows [34]

(8) $$F\left(x\right)=\frac{1}{T}\sum_{t=1}^{T}f_{t}\left(x\right),$$

where ***x*** denotes a group of normalized input features, *f_t_*(·) represents the trained model part after the *t*-th trial, *T* is the number of trials, and *F*(·) represents the final outputs. After applying the inverse normalization to the final outputs, we can obtain the final prediction results, i.e., the final predicted OVFR, GVFR and WVFR. In the subsequent sections, we focus on the predictive results and effects of the CNN-based model with ensemble learning.

## 4. Experimental Results and Discussion

To evaluate the prediction performance of the proposed CNN-based model, we selected the following criteria for each phase

Root mean squared error (RMSE)

(9) $$\mathrm{RSME}=\sqrt{\frac{1}{N}\sum_{i=1}^{N}{\left({\hat{q}}_{i}-q_{i}\right)}^{2}},$$

Coefficient of determination (*R*^2^)

(10) $$\left\{\begin{array}{l} R^{2}=1-\frac{\sum_{i=1}^{N}{\left(q_{i}-{\hat{q}}_{i}\right)}^{2}}{\sum_{i=1}^{N}{\left(q_{i}-{\bar{q}}_{i}\right)}^{2}}\\ \bar{q}=\frac{1}{N}\sum_{i=1}^{N}q_{i} \end{array}\right.,$$

Mean absolute percentage error (MAPE)

(11) $$\mathrm{MAPE}=\frac{1}{N}\sum_{i=1}^{N}\left|\frac{{\hat{q}}_{i}-q_{i}}{q_{i}}\right|\cdot 100\%,$$

Mean quoted error (MQE) [13]

(12) $$\mathrm{MQE}=\frac{1}{N}\sum_{i=1}^{N}\frac{\left|{\hat{q}}_{i}-q_{i}\right|}{q_{max}}\cdot 100\%,$$

where *N* is the sample size; ${\hat{q}}_{i}$ denotes the *i*-th predicted volume flow rate of one individual phase, i.e., ${\hat{Q}}_{o}$, ${\hat{Q}}_{g}$ or ${\hat{Q}}_{w}$; $q_{i}$ denotes the *i*-th reference volume flow rate of one individual phase, i.e., *Q_o_*, *Q_g_* or *Q_w_*; $\bar{q}$ represents the mean value of $q_{i}$ and $q_{max}$ represents the maximum of all reference volume flow rates of one individual phase.

**Figure 6:**
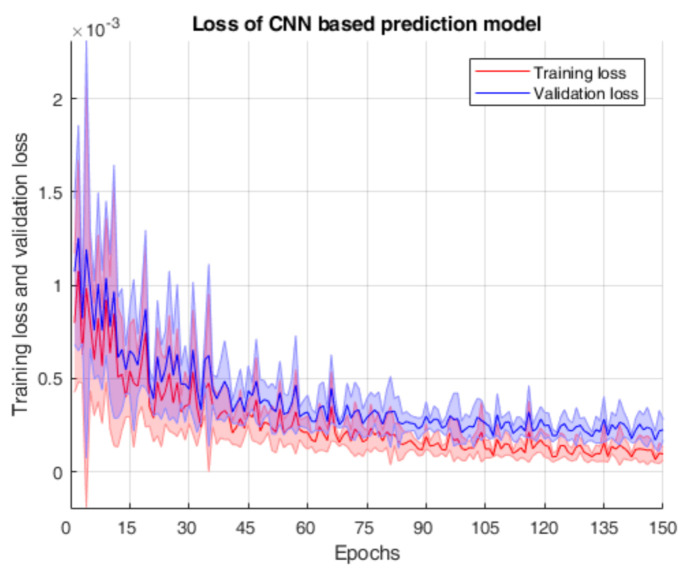
Loss curves of the CNN-based prediction model over 5 trials. The solid lines and the shaded areas represent the mean and the standard deviation over 5 trials, respectively.

The hyper-parameters used to train the model are chosen as shown in Table 4, through trial and error. In this paper, the model’s architecture was implemented by PyTorch, which is a prevailing open-source deep-learning platform [35], and the training process runs on 4 NVIDIA GeForce GTX 1080 Ti GPUs. After training, we can obtain the CNN-based prediction model to predict the volume flow rates of the oil-phase, the gas-phase and the water-phase simultaneously. The corresponding loss curves over five trials are shown in Figure 6.

From Figure 6, it is found that the training loss and the validation loss reduce relatively significantly in the first 60 training epochs, and the training loss curve is below the validation loss curve when the loss curves converge, which satisfies our expectation. In addition, we can see that the model does not suffer overfitting. Hence, it is feasible to realize the volume flow rate predictions for the oil, gas and water using the proposed CNN-based model.

After training and validation, for each individual phase, the defined criteria of the CNN-based prediction model over five trials on different datasets are evaluated and shown as Table 5. In this table, there are three flow phases and three types of dataset, and mean values and standard deviation (STD) values over five trials illuminate the magnitude and the fluctuation of each criterion, respectively. Note that RMSE, MAPE and MQE are better when closer to 0.0, while *R*^2^ is better when closer to 1.0. These criteria are utilized to evaluate the proposed model but not to train the model and reflect the prediction performance of the proposed CNN-based model. It is found from Table 5 that, for the same type of dataset and criterion, the prediction performances are relatively close between the oil-phase and the water-phase, and the prediction performance for the gas-phase is slightly inferior to those for the other two flow phases.

We also compared the prediction results of the CNN-7-based model with those in [18] and summarized the results in Table 6. From this table, we can see that the proposed CNN-7-based model can obtain better MAPE than the CNN_dual_ model in [18], which demonstrates the superiority of the proposed model in this paper and is in accordance with the analyses in Section 3.2.

**Figure 7:**
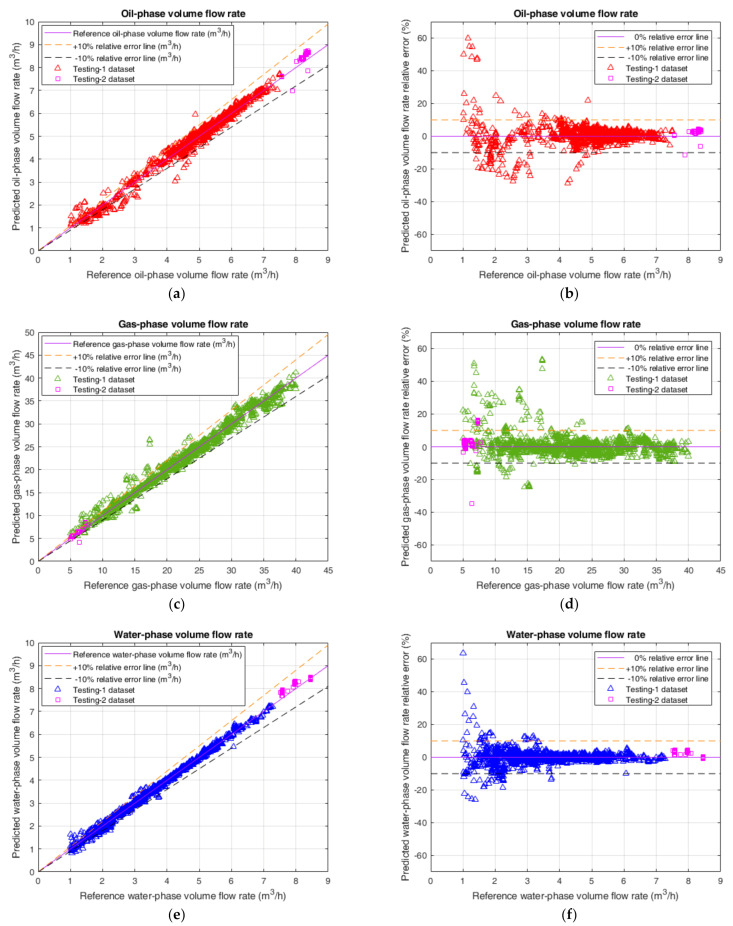
The predicted results on the testing-1 and testing-2 datasets of the proposed CNN-based volume flow rate prediction model combined with ensemble learning. (**a**) The volume flow rate prediction of the oil-phase; (**b**) The prediction relative error of the oil-phase; (**c**) The volume flow rate prediction of the gas-phase; (**d**) The prediction relative error of the gas-phase; (**e**) The volume flow rate prediction of the water-phase; (**f**) The prediction relative error of the water-phase.

Figure 7 shows the predicted results including the volume flow rate predictions and the corresponding relative errors on the testing-1 and testing-2 datasets for the oil-phase, the gas-phase and the water-phase. From Figure 7, it can be seen that, for the testing-1 dataset and the testing-2 dataset, the datapoints of all three individual flow phases are predominantly located in the ±10% relative error band, which illustrates that the predictive effect of the CNN-7 prediction model combined with ensemble learning is remarkable.

Based on the results in Figure 7, the quantitative statistics about the performance related to CNN-7 for three different flow phases on the testing-1 dataset are calculated and summarized in Table 7. In this table, ${\left|\xi \right|}_{e}$ denotes the proportion of datapoints within ±*e*% relative error band to the total datapoints, which can be considered as the confidence level of relative errors within ±*e*%. The corresponding formula is shown as below

(13) $${\left|\xi \right|}_{e}=\frac{\sum_{i=1}^{N}I\left\{\left|\xi_{i}\right|\;\leq e\right\}}{N}\cdot 100\%,$$

where *ξ_i_* denotes the *i*-th predicted relative error expressed as a percentage of one individual flow phase; *e* denotes the selected error bound expressed as a percentage which is a positive value; *N* represents the number of total datapoints of the dataset; *I*$\left\{\left|\xi_{i}\right|\;\leq e\right\}$ represents the indicator function, i.e., when $\left|\xi_{i}\right|\;\leq e$ it is equal to 1 and when $\left|\xi_{i}\right|\;>e$ it is equal to 0 [36].

From the statistics in Table 7, it can be seen that the confidence levels between the oil and the water are relatively close, and the confidence level of the gas is inferior to those of the other two phases, which is consistent with the aforementioned discussion obtained from Table 5. Meanwhile, it is found that ${\left|\xi \right|}_{10}$ of all three flow phases is greater than 90%, which demonstrates that the prediction performance of our proposed CNN-based model is impressed and the predictive effects for three different flow phases are all fairly good. Hence, it is feasible to utilize this model in practical applications for individual phase-volume flow-rate measurement of the oil–gas–water three-phase intermittent flow.

As shown in Table 8, we also provide statistics about the time consumption of the training and the evaluation for the proposed CNN-based model, which include the training time with 4 NVIDIA GeForce GTX 1080 Ti GPUs and the evaluation time with 1 Intel Xeon CPU E5-2678 v3 or 1 NVIDIA GeForce GTX 1080 Ti GPU. It is notable that the evaluation process is aimed at one testing sample of the testing-1 dataset and can be considered as the practical working condition of the proposed model after training and deploying. In the practical real-time application scenario, after one sample is collected, it can be fed into the proposed trained CNN-based model to obtain the volume flowrates of oil, gas and water. From the statistics in Table 8, it is found that, although the training process of the model is relatively time-consuming, the evaluation process only takes 0.43 s on a CPU and 0.01 s on a GPU, which demonstrates that the real-time measurement ability of the proposed model after training and deploying is satisfactory.

In our model, we used eight types of measurement data as input parameters, which are *T*, *P*, Δ*P*_1_, Δ*P*_2_, *β*_1_, *A*_1_, *β*_2_ and *A*_2_. As a contrast, we reduced the number of input parameters to 5 (i.e. *T*, *P*, Δ*P*_1_, *β*_1_ and *A*_1_). We trained the models using the same hyper-parameters as shown in Table 4 and obtained Table 9 to show RMSE, MAPE and ${\left|\xi \right|}_{10}$ on the testing-1 dataset with different input parameters. From this table, it is found that the prediction performances of the model using eight input parameters are much better than those of the model using five input parameters. In addition, we also find that there is no obvious difference in the training time and the evaluation time between these two situations. Hence, these eight types of measurement data are needed to obtain the individual volume flow rates of the oil-phase, the gas-phase and the water-phase effectively.

Furthermore, we can calculate RMSE, MAPE and ${\left|\xi \right|}_{10}$ on the testing-2 dataset for different flow phases using the trained model to verify the generalization ability of the proposed model. The relevant results are summarized as Table 10. It can be found that the predictive effect is pretty good for the prediction on the testing-2 dataset, which verifies that the trained CNN-based model can be used to predict the flow rate out of the range of the experimental dataset (shown as the blue points in Figure 3) to some degree. This is capable of demonstrating the generalization ability of our proposed CNN-based model from the aspect of practical application.

**Figure 8:**
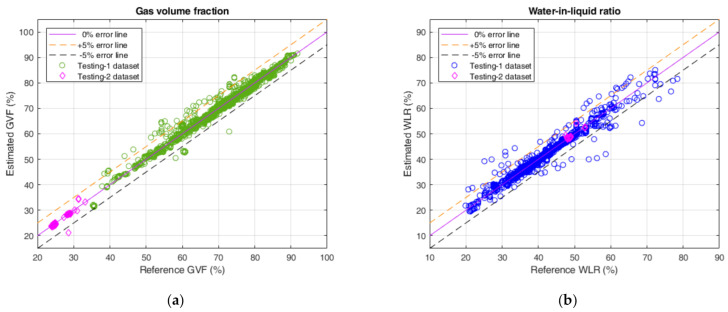
The estimation of GVF and WLR with the prediction results on the testing-1 and testing-2 datasets obtained by the proposed CNN-based model. (**a**) The estimation of GVF; (**b**) The estimation of WLR.

In addition, after obtaining the volume flow rates of three individual phases, we can also estimate GVF and WLR with the following formulas

(14) $${\mathrm{GVF}}_{estimation}=\frac{{\hat{Q}}_{g}}{{\hat{Q}}_{o}+{\hat{Q}}_{g}+{\hat{Q}}_{w}}\cdot 100\%,$$

(15) $${\mathrm{WLR}}_{estimation}=\frac{{\hat{Q}}_{w}}{{\hat{Q}}_{o}+{\hat{Q}}_{w}}\cdot 100\%,$$

where the meanings of ${\hat{Q}}_{o}$, ${\hat{Q}}_{g}$ and ${\hat{Q}}_{w}$ have been mentioned above. Figure 8 shows the estimation results of GVF and WLR using the predicted volume flow rates on the testing-1 and testing-2 datasets. It can be seen that the estimated GVF and the estimated WLR are almost in the ±5% error band. Quantitatively, Table 11 shows that the proportions of estimated GVF and WLR within the ±5% error band are all greater than 95% for the testing-1 dataset and the testing-2 dataset. The aforementioned results are remarkable and demonstrate the powerful ability of our proposed CNN-based volume flow rate prediction model.

**Figure 9:**
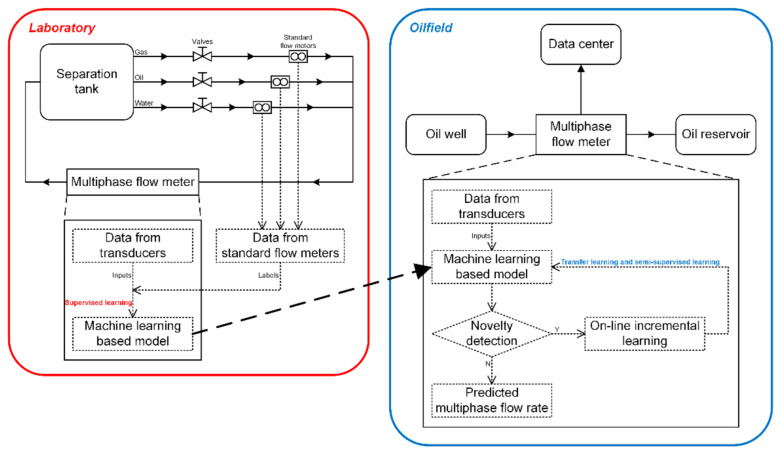
The machine-learning-based data analytics framework for measurement of multiphase flow.

In the aforementioned discussions, we focused on the supervised learning regarding the transducer data from the laboratory. In our proposed method, we can simultaneously obtain the volume flow rates of the oil-phase, the gas-phase and the water-phase of the oil–gas–water three-phase intermittent flow using the CNN-based prediction model. From the perspective of practical applications, an issue that needs to be considered is the adaptability of the proposed model to the real well-head field data. Hence, we put forward, in Figure 9, a possible novel machine-learning-based data analytics framework for the measurement of multiphase flow in the petroleum industry. In this framework, firstly, a laboratory set-up, on which a series of experiments to reveal the characterization of the multiphase flow to be studied can be conducted, is the basis of the framework and is also the research focus of this paper. The multiphase flow meter to be developed is installed on the test section of the set-up and data from the transducers used in the multiphase flow meter can be sampled. Meanwhile, the labeled data that stand for the true flow conditions can be sampled from standard flow meters installed on the single-phase flow pipelines of the set-up. Supervised learning is used to model the transducer data in the laboratory to obtain the needed predicted flow rate. Then, we can deploy the supervised learning trained model into the on-site multiphase flow meter in the oilfield to predict the multiphase flow rate. By using novelty detection [37,38], the model needs to be updated with on-line incremental learning if necessary [39]. In this framework, the novelty detection is supposed to work together with transfer learning and semi-supervised learning after the model trained by using the laboratory data is deployed in the practical oilfield. It detects whether the laboratory-data-based training model is satisfactory when applied in the field. If not, it will trigger that a separator-based mobile metering vehicle to sample a batch of labeled data and use them to update the model by using transfer learning and semi-supervised learning [16,19,40,41,42], which will be explored in detail in our future works. Considering the difference and correlation of the transducer data between the laboratory and the oilfield, transfer learning techniques can be introduced to update the model. Due to the scarcity of the labeled data in the oilfield, semi-supervised learning can be also used to improve the predictive effect further.

At present, the measuring working pressure ranges from 123.14 to 611.43 kPa in our laboratory, as shown in Table 2. Based on the data analytics framework, the model trained from the laboratory data via supervised learning can be used as the pre-trained model in the actual oilfield. In the higher-pressure applications of the actual oilfield, the pre-trained model needs to be corrected and fine-tuned using some transfer learning and semi-supervised learning techniques.

It is worth pointing out that one drawback of the model based on deep learning is its interpretability. This is almost the same issue that all deep-learning techniques have to face to currently. Although deep learning has achieved great successes in different fields in the past few years, the explanation of why deep learning works and achieves good performances is still an open problem in machine learning. Different deep-learning techniques may have different performances for different problems. Although there is no evidence that CNN must be universally superior in all fields, the CNN-based prediction model achieves attractive and satisfactory results in our study about the volume flow rate measurement of the multiphase flow. In addition, the CNN-based model is flexible, so it is convenient to introduce transfer learning and semi-supervised learning into the multiphase flow measurement field with the CNN-based model as the underlying architecture in the future works, which is capable of bringing new data analytics into the multiphase flow measurement and expanding the innovative application scenarios of deep learning.

Moreover, from the flow regime map, shown as Figure 3, it can be seen that there is currently a mass of slug flow and a little elongated bubble flow in our collected experimental data. Due to the limitations of our current experimental flow loop system, the flow regimes that we can acquire are indeed finite, i.e., we can only obtain the intermittent flow (including the slug flow and the elongated bubble flow) effectively. In the future, after improving the experimental flow loop system further, we can obtain more flow regimes so that more abundant experimental data can be obtained to help train the more general prediction model, which can effectively realize measurement in various flow regimes.

## 5. Conclusions

In this paper, we propose a deep-learning-based measurement method, i.e., the CNN-based model. For predicting the volume flow rates of the oil-phase, the gas-phase and the water-phase in the oil–gas–water three-phase intermittent flow simultaneously, the CNN-based prediction model is used to analyze the measurement data including the flow temperature, the pipeline pressure, the differential pressures from the convergent section and divergent section of the Venturi tube and the phases and amplitudes of microwaves. To acquire sufficient data, we carried out various experiments on a test loop pipeline in the 50 mm diameter, in which GVF and WLR ranges are 23.77–94.45% and 14.95–86.97%, respectively, and the gas flow superficial velocity range is 0.66–5.23 m/s and the liquid flow superficial velocity range is 0.27–2.14 m/s.

After comprehensive analyses, we find that the proposed CNN-7 can simultaneously predict the volume flow rates of three different flow phases of the oil–gas–water three-phase intermittent flow. The predictive effects for the oil-phase and the water-phase are close, while the predictive effect for the gas-phase is slightly inferior to those for the other two flow phases. However, on the whole, for the three different flow phases, the prediction effects are all fairly good. When combined with ensemble learning, the model can obtain satisfactory final predictive results and effect. On the testing-1 dataset, for the volume flow rates of the oil-phase, the gas-phase and the water-phase, the proposed CNN-based prediction model can provide the relative errors within ±5% with 84.62%, 79.55% and 87.31% confidence levels, and within ±10% with 94.49%, 92.56% and 95.71% confidence levels. On the testing-2 dataset, the trained model can also obtain pretty good prediction results, which shows the generalization ability of the model. In addition, the consuming time of a prediction with one sample on an Intel Xeon CPU E5-2678 v3 and an NVIDIA GeForce GTX 1080 Ti GPU are 0.43 and 0.01 s, respectively. These discussions demonstrate that the proposed model can meet the practical industrial application demands for the real-time flow rate measurement of the oil–gas–water three-phase intermittent flow. Compared with the expensive commercialized MPFMs, the experimental results also illuminate that the proposed CNN-based model can implement convenient, cost-effective and accuracy-assured multiphase flow rate measurement for an individual well with low liquid-phase production in the petroleum industry.

As we know, the flow characteristics of the multiphase flow are very complex, so finding a relevant real-time measurement technique is still a big challenge and an open problem. This paper proposes the CNN-based model with ensemble learning to implement the data-driven black-box soft-sensing from multiple sensors for the flow rate prediction of the oil–gas–water three-phase intermittent flow, and the obtained results are convincible and satisfactory, which quantitatively verifies the feasibility and validity of the proposed model in individual well measurement in the petroleum industry. In addition, the CNN-based model is fairly suitable to be the underlying architecture of some transfer learning methods and semi-supervised learning methods, and the introduction of transfer learning and semi-supervised learning can enhance the practicability of the proposed prediction model. This paper effectively demonstrates the potential and promising prospect of utilizing the deep learning techniques in multiphase flow measurement and, to some extent, lays the foundation for future works, which can introduce new data analytics into the multiphase flow measurement field and enrich the application areas of deep learning.

## Figures and Tables

**Table 1:** Description of standard flow meters.

Measuring Meter	Measuring Range
Standard gas flow meter (thermal flow meter)	0–90 m^3^/h
Standard oil flow meter (dual rotor flow meter)	0.6–36 m^3^/h
Standard water flow meter (electromagnetic flow meter)	0.17–26 m^3^/h

**Table 2:** Working conditions of the experiments.

Experimental Parameters	Range
*T* (°C)	29.6–41.06
*P* (kPa)	123.14–611.43
Δ*P*_1_ (kPa)	0.12–62.1
Δ*P*_2_ (kPa)	0–31.74
*β*_1_ (°)	70.45–180
*A*_1_ (dB)	−0.67–3.74
*β*_2_ (°)	63.93–180
*A*_2_ (dB)	−4.08–3.42
Oil volume flow rate (m^3^/h)	1–8.39
Gas volume flow rate (m^3^/h)	5.01–40
Water volume flow rate (m^3^/h)	1–8.46
Gas superficial velocity (m/s)	0.66–5.23
Liquid superficial velocity (m/s)	0.27–2.14
Gas volume fraction (GVF) (%)	23.77–94.45
Water-in-liquid ratio (WLR) (%)	14.95–86.97

**Table 3:** Architecture of CNN-7 for the volume flow rate predictions.

Layer Name	Structure	Output Shape
CONV1	10 × 8, 896, s = 2, p = 0; BN; ReLU	896 × 896 × 1
	Permute the dimensions of the output	1 × 896 × 896
CONV2	3 × 3, 32, s = 2, p = 1; BN; ReLU	32 × 448 × 448
CONV3	3 × 3, 64, s = 2, p = 1; BN; ReLU	64 × 224 × 224
CONV4	3 × 3, 64, s = 1, p = 1; BN; ReLU	64 × 224 × 224
CONV5	3 × 3, 128, s = 2, p = 1; BN; ReLU	128 × 112 × 112
CONV6	3 × 3, 256, s = 2, p = 1; BN; ReLU	256 × 56 × 56
	Average-pooling, 56 × 56	256 × 1 × 1
	Squeeze the dimensions of the output	256 × 1
FC	Linear	3 × 1

**Table 4:** Hyper-parameters for the model training.

Hyper-Parameters	Setting
Number of trials *T*	5
Number of epochs *E*	150
Batch size *B*	64
Learning rate *λ*	0.001

**Table 5:** Criteria for different datasets over 5 trials for different flow phases using the CNN-based prediction model.

Flow Phase	Dataset	RMSE (m^3^/h)		*R* ^2^		MAPE (%)		MQE (%)	
		Mean	STD	Mean	STD	Mean	STD	Mean	STD
Oil	Training dataset	0.1294	0.0153	0.9914	0.0020	2.5741	0.2933	0.5131	0.0880
	Validation dataset	0.1753	0.0159	0.9828	0.0032	3.2951	0.2651	0.6941	0.0774
	Testing-1 dataset	0.1945	0.0108	0.9787	0.0024	3.6488	0.2129	0.7535	0.0609
Gas	Training dataset	0.5834	0.0899	0.9945	0.0016	2.3290	0.3709	0.7589	0.1197
	Validation dataset	1.0539	0.0782	0.9824	0.0026	3.6297	0.3001	1.1992	0.1036
	Testing-1 dataset	1.1671	0.0445	0.9801	0.0015	4.3257	0.2136	1.3975	0.0740
Water	Training dataset	0.0962	0.0128	0.9959	0.0011	2.5060	0.3158	0.3838	0.0587
	Validation dataset	0.1162	0.0114	0.9946	0.0011	2.9345	0.2715	0.4630	0.0470
	Testing-1 dataset	0.1214	0.0102	0.9929	0.0012	3.0969	0.2655	0.4661	0.0447

RMSE, *R*^2^, MAPE and MQE are illuminated as Equations (9)–(12).

**Table 6:** MAPE comparison between CNN_dual_ model in [18] and CNN-7-based model in this paper.

Model Type	Flow Phase	MAPE (%)
CNN_dual_ model in [18]	Oil	5.30
	Gas	−
	Water	3.95
CNN-7-based model in this paper	Oil	3.65
	Gas	4.33
	Water	3.10

**Table 7:** ${\left|\xi \right|}_{e}$ on the testing-1 dataset for different flow phases using the CNN-based prediction model.

Flow Phase	${\left|\xi \right|}_{5}$	${\left|\xi \right|}_{10}$
Oil	84.62%	94.49%
Gas	79.55%	92.56%
Water	87.31%	95.71%

**Table 8:** Time consumption of the training and the evaluation process.

Process Type		Time
Training process	4 GPUs	5.01 h
Evaluation process	1 CPU	0.43 s
	1 GPU	0.01 s

**Table 9:** RMSE, MAPE and ${\left|\xi \right|}_{10}$ on the testing-1 dataset with different input parameters.

Number of Input Parameters	Flow Phase	RMSE (m^3^/h)	MAPE (%)	${\left|\xi \right|}_{10}$
5 (*T*, *P*, Δ*P*_1_, *β*_1_, *A*_1_)	Oil	0.2776	5.0613	90.96%
	Gas	1.5111	6.2232	87.18%
	Water	0.1781	4.3006	92.69%
8 (*T*, *P*, Δ*P*_1_, Δ*P*_2_, *β*_1_, *A*_1_, *β*_2_, *A*_2_)	Oil	0.1945	3.6488	94.49%
	Gas	1.1671	4.3257	92.56%
	Water	0.1214	3.0969	95.71%

**Table 10:** RMSE, MAPE and ${\left|\xi \right|}_{10}$ on the testing-2 dataset for different flow phases using the CNN-based prediction model.

Flow Phase	RMSE (m^3^/h)	MAPE (%)	${\left|\xi \right|}_{10}$
Oil	0.2731	2.9881	98.57%
Gas	0.4630	3.9616	88.57%
Water	0.2554	3.0986	100%

**Table 11:** Proportion within ±5% error band for GVF and WLR estimation on the testing-1 and testing-2 datasets.

Dataset	Phase Fraction	Proportion within ±5% Error Band
Testing-1 dataset	GVF*_estimation_*	96.86%
	WLR*_estimation_*	97.18%
Testing-2 dataset	GVF*_estimation_*	98.57%
	WLR*_estimation_*	100%

## Data Availability

The data presented in this study are available on request from the corresponding author. The data are not publicly available due to privacy and future works.
